# Remifentanil to Maintain Unresponsiveness While Propofol Concentrations Decline Before Emergence in Two Patients With Prior Emergence Agitation

**DOI:** 10.1155/cria/9359809

**Published:** 2026-11-01

**Authors:** Jeffrey D. Swenson, G. Colton Gardner, Ken B. Johnson, Evelyn C. Loose, Alastair Eric Moody

**Affiliations:** ^1^ Department of Anesthesiology University of Utah Salt Lake City Utah USA utah.edu; ^2^ Department of Anesthesiology The University of Arizona College of Medicine 1501 N. Campbell Ave. Tucson 85724 Arizona USA arizona.edu

**Keywords:** anesthesia, emergence delirium, intravenous, pharmacokinetics, propofol, recovery room, remifentanil

## Abstract

**Background:**

Emergence agitation is an unpredictable and potentially harmful state during recovery from general anesthesia. We describe a pharmacokinetic strategy intended to permit the propofol concentration to decline further before emergence while maintaining unresponsiveness with remifentanil.

**Case:**

Two adults experienced severe emergence agitation after propofol‐remifentanil total intravenous anesthesia. During subsequent procedures, propofol was discontinued well before emergence, and remifentanil was continued or administered intermittently while estimated propofol effect‐site concentrations declined. Both patients subsequently emerged calmly without observed hemodynamic or ventilatory compromise.

**Conclusions:**

These findings are hypothesis‐generating. They suggest that separating the decline in propofol concentration from the time of awakening warrants prospective study as a strategy to reduce emergence agitation in patients with a prior episode. Modeled rather than measured concentrations, uncontrolled clinical factors, and the use of large remifentanil boluses limit any causal or dosing conclusions.

## 1. Introduction

Emergence agitation (EA) is a transient cognitive state characterized by confusion and erratic movement during anesthetic recovery [1]. Patients affected by EA may suffer injury or compromised surgical outcomes [2]. Although several patient, anesthetic, and surgical factors are associated with EA, predicting its occurrence remains challenging [3]. Existing treatments, including dexmedetomidine, do not consistently prevent or treat this disorder [4, 5].

We describe two patients with markedly different emergence profiles during sequential anesthetics. Both developed severe EA after propofol‐remifentanil total intravenous anesthesia (TIVA). During subsequent procedures, the anesthetic technique was modified to reduce the estimated propofol effect‐site concentration (Ce) before emergence while maintaining unresponsiveness with remifentanil. Both patients subsequently emerged calmly and were discharged without observed complications during the procedures in which this approach was used.

Titration was informed by a remifentanil‐propofol interaction model of unresponsiveness [6], which identifies Ce pairs with similar predicted effects: low‐remifentanil/high‐propofol and high‐remifentanil/low‐propofol combinations may produce similar probabilities of unresponsiveness. After propofol discontinuation, remifentanil was continued or administered intermittently while the estimated propofol Ce declined below 1.0 μg/mL [7, 8].

The University of Utah Institutional Review Board Case Report Using Existing Data Author Worksheet was completed and indicated that submission to the IRB was not required. Both patients provided verbal consent for publication. No direct patient identifiers are included in this report.

## 2. Case Presentation

### 2.1. Case 1

A 22‐year‐old man (178 cm, 80 kg) with a history of attention‐deficit/hyperactivity disorder, autism spectrum disorder, cannabis use, and tobacco use presented for tendon repair of the right small finger. He agreed to general anesthesia with a peripheral nerve block for postoperative analgesia.

Ten minutes before transfer, the patient received intravenous midazolam 2 mg and remained calm and cooperative. Anesthesia was induced with fentanyl 100 μg and propofol 150 mg, and a supraglottic airway was placed. Maintenance was provided with propofol 0.1 mg/kg/min and remifentanil 0.2 μg/kg/min. He also received dexamethasone 4 mg and ondansetron 4 mg.

Surgery was completed uneventfully after 45 min, and both infusions were discontinued. A digital block using 7 mL of 0.25% bupivacaine was performed before splint placement. Approximately 8 min after discontinuation, the patient opened his eyes on command and breathed spontaneously. Figure 1 presents simulated propofol and remifentanil Ce values at emergence using published pharmacokinetic models [6–8].

**FIGURE 1:**
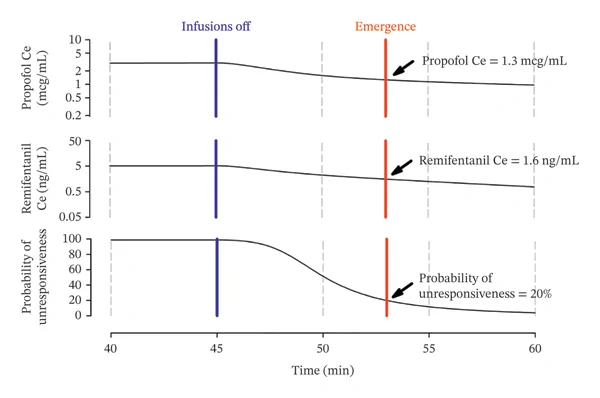
Simulated propofol and remifentanil effect‐site concentrations (Ces) for the first anesthetic of case 1 based on published pharmacokinetic models (top two panels). Propofol and remifentanil infusions of 0.1 mg/kg/min and 0.2 μg/kg/min, respectively, were stopped at 45 min (blue line). The red line indicates the propofol and remifentanil Ce at emergence from anesthesia. The bottom panel presents the probability of patient unresponsiveness based on a remifentanil‐propofol interaction model. Emergence occurred 8 min after stopping infusions at propofol and remifentanil Ces of 1.3 μg/mL and 1.6 ng/mL, respectively.

The supraglottic airway was removed, and the patient was transported to the postanesthesia care unit (PACU), where he became combative, attempted to leave the gurney, and struck staff. Verbal redirection was unsuccessful. He required four‐point restraints and received dexmedetomidine 20 μg, haloperidol 10 mg in divided doses, and midazolam 6 mg in divided doses over 15 min, with continuous monitoring of oxygen saturation and respiratory rate. He remained somnolent but arousable. After approximately 3 h, his affect normalized. An ultrasound‐guided selective ulnar nerve block was performed at the forearm using 5 mL of 0.5% bupivacaine, and he was discharged without further incident.

Three months later, he returned for a second‐stage procedure and declined monitored anesthesia care because he wished to be fully asleep for the procedure. General anesthesia was therefore planned, with a peripheral nerve block for postoperative analgesia.

For the second anesthetic, midazolam 2 mg was given preoperatively, and the patient remained cooperative during an ultrasound‐guided selective ulnar nerve block at the mid‐forearm using 5 mL of 0.5% bupivacaine. Anesthesia was induced with fentanyl 100 μg and propofol 150 mg. Rocuronium 50 mg facilitated tracheal intubation given the anticipated duration. Sugammadex 200 mg was administered for reversal, which was confirmed with neuromuscular monitoring. Maintenance and antiemetic prophylaxis were unchanged.

Thirty minutes before completion of surgery and splint placement, the propofol and remifentanil infusions were discontinued after a total infusion time of 126 min. Remifentanil boluses were administered to maintain unresponsiveness while the estimated propofol Ce declined below 1.0 μg/mL (Figure 2). Based on the interaction model [6], a remifentanil Ce target of 20 ng/mL was used. The patient received remifentanil 200 μg (2.5 μg/kg) at 5 and 10 min, followed by 300 μg (3.75 μg/kg) at 15, 20, and 25 min. No changes in vital signs, tidal volume, or peak airway pressure were observed. Thirty‐five minutes after propofol discontinuation, the patient awakened, followed commands, was extubated, and was transported to the PACU. Emergence was calm, and he was discharged 33 min after arrival.

**FIGURE 2:**
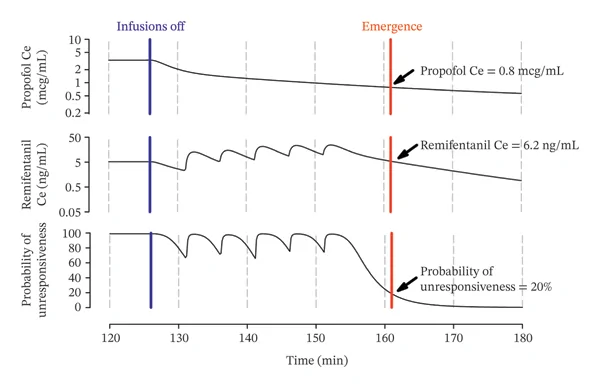
Simulated propofol and remifentanil effect‐site concentrations (Ces) for the second anesthetic of case 1 based on published pharmacokinetic models (top two panels). Propofol and remifentanil infusions of 0.1 mg/kg/min and 0.2 μg/kg/min, respectively, were stopped at 126 min (blue line). After stopping the propofol infusion, the patient received intermittent remifentanil boluses (200–300 μg) to maintain unresponsiveness while propofol Ce decreased below 1.0 μg/mL. The red line indicates the propofol and remifentanil Ces at emergence from anesthesia. The bottom panel presents the probability of unresponsiveness based on remifentanil‐propofol interaction models. Emergence occurred 35 min after stopping infusions at propofol and remifentanil concentrations of 0.8 μg/mL and 6.2 ng/mL, respectively.

### 2.2. Case 2

A 49‐year‐old man (89 kg; body mass index, 26.6 kg/m^2^) with no significant medical history presented for right quadriceps tendon repair.

The patient received midazolam 2 mg before transfer. In the operating room, anesthesia was induced with propofol 200 mg and fentanyl 200 μg. General anesthesia was maintained using a single‐syringe TIVA technique containing propofol 10 mg/mL and remifentanil 20 μg/mL, with propofol started at 90 μg/kg/min.

Both propofol and remifentanil were discontinued after a total infusion duration of 60 min, and the supraglottic airway was removed 5 min later. Following emergence, the patient developed severe agitation requiring physical restraint by six staff members for several hours in the PACU.

Several months later, he returned for left quadriceps tendon repair, and the emergence strategy was modified.

Induction included the same medications used during the first procedure. Propofol was maintained at 90 μg/kg/min and remifentanil at 0.2 μg/kg/min for 42 min. Propofol was discontinued 25 min before completion of surgery. Remifentanil was continued and then administered as intermittent boluses totaling 3 mg to maintain unresponsiveness while the estimated propofol Ce declined.

Electroencephalographic monitoring demonstrated Patient State Index values of 34 and 32 at 22 min before extubation, 34 at 17 min before extubation, and 30 at 5 min before extubation, with transient decreases to 25 after bolus administration. From propofol discontinuation to extubation, noninvasive systolic blood pressure ranged from 108 to 123 mmHg and mean arterial pressure from 73 to 93 mmHg (70–85 mmHg during maintenance before propofol discontinuation), peripheral oxygen saturation from 98% to 99%, and end‐tidal carbon dioxide from 35 to 38 mmHg. No changes in tidal volume or peak airway pressure were observed.

The procedure concluded after 67 min, and the patient was extubated 3 min later. Emergence was calm, the patient followed commands, and no agitation was observed. He was transported to the PACU and discharged without incident.

## 3. Discussion

The ability to conduct a controlled recovery from anesthesia is important in patients at risk for EA. More broadly, patient safety may be compromised when agitation occurs during emergence [2]. There is no consensus on optimal prevention or treatment strategies [9]. Remifentanil‐based approaches have been evaluated in pediatric and adult populations [10, 11], while pharmacologic approaches such as dexmedetomidine have produced inconsistent results [4, 5].

EA is likely multifactorial and may be influenced by patient characteristics, medications, pain, and environmental factors. Accordingly, the different emergence profiles observed in these cases cannot be attributed with certainty to a single pharmacologic variable.

Medications common to both anesthetics included midazolam, fentanyl, remifentanil, and propofol. Evidence on the contribution of each drug to EA specifically is limited. The studies below examined related but distinct outcomes, postoperative delirium and postoperative cognitive dysfunction, in different populations, and therefore provide only indirect context for these cases. Midazolam premedication was not associated with early postoperative delirium in one study of older adults [12], and prior exposures to midazolam were well tolerated in the first patient. Remifentanil did not reduce postoperative cognitive dysfunction compared with fentanyl in a separate comparative study [13]. Postoperative delirium was more frequent with propofol than with dexmedetomidine sedation in older adults undergoing orthopedic surgery [14]. Although this finding concerns delirium rather than EA, it raises the possibility that residual propofol Ces contributed to the agitation observed here.

Pain was considered as a contributor during the first anesthetic in Case 1. A digital block using 0.25% bupivacaine was performed at the conclusion of surgery, and an ultrasound‐guided selective ulnar nerve block was performed in the PACU. Although inadequate analgesia cannot be excluded, the patient demonstrated no overt distress before agitation.

During the first anesthetic in Case 1, responsiveness occurred 8 min after discontinuation of anesthesia at an estimated propofol Ce of 1.3 μg/mL and remifentanil Ce of 1.6 ng/mL. These values were modeled rather than measured. Processed electroencephalographic monitoring was not used during either anesthetic in Case 1, so these estimates could not be compared with an independent measure of hypnotic depth. During the second anesthetic, propofol and remifentanil were discontinued 30 min before the anticipated time of extubation after a total infusion time of 126 min. Remifentanil boluses of 200 μg at 5 and 10 min, followed by 300 μg at 15, 20, and 25 min, were administered to maintain unresponsiveness while the estimated propofol Ce declined. Thirty‐five minutes after propofol discontinuation, the patient awakened and was extubated at an estimated propofol Ce of 0.8 μg/mL and remifentanil Ce of 6.2 ng/mL. The remifentanil‐propofol interaction surface and the estimated emergence points for Case 1 are shown in Figure 3. The interaction model used to inform management has not been validated for anesthetic emergence and provides only a framework for interpreting these observations. The model also does not account for the residual effect of fentanyl given at induction in both patients, which may have contributed to the overall opioid effect and probability of unresponsiveness at emergence.

**FIGURE 3:**
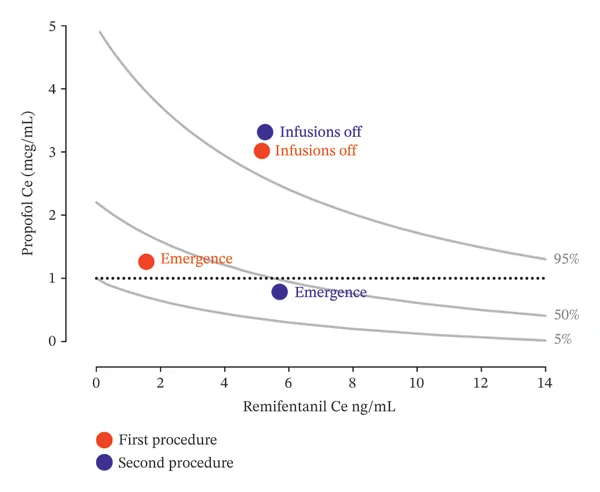
Schematic of the remifentanil‐propofol interaction model of probability of unresponsiveness for case 1. Grey lines represent the 5%, 50%, and 95% iso‐effect lines for predicted remifentanil‐propofol effect‐site concentration pairs. Filled circles indicate the propofol‐remifentanil Ce pairs at the time infusions were stopped and at emergence for each procedure. Propofol Ces at emergence were 1.3 μg/mL (first procedure) and 0.8 μg/mL (second procedure), illustrating that the modified emergence strategy resulted in a lower estimated propofol effect at awakening.

A similar strategy was used in Case 2. Propofol was discontinued approximately 25 min before the end of surgery, and remifentanil was administered as intermittent boluses totaling 3 mg to maintain unresponsiveness while the estimated propofol Ce declined. Patient State Index values ranged from 30 to 34 before bolus administration, with transient decreases to 25 after boluses, without observed changes in tidal volume or peak airway pressure. Emergence was calm and without observed agitation.

In both patients, calm emergence during the subsequent procedure occurred after the estimated propofol Ce had declined below 1.0 μg/mL, whereas emergence at an estimated propofol Ce of 1.3 μg/mL was associated with agitation in Case 1. Pharmacokinetic models cannot determine exact Ces in individual patients, and Case 2 did not provide a direct modeled comparison. These observations therefore do not establish a propofol threshold or a causal relationship.

The approach differed from strategies based on increasing longer‐acting sedation. Remifentanil was used as a rapidly metabolized adjunct while the estimated propofol Ce declined. Its synergy with propofol may have contributed to maintenance of unresponsiveness. Remifentanil was administered intermittently, although a continuous infusion could be studied. Its rapid offset permitted communication soon after the final dose in these two cases.

Although large remifentanil boluses were administered, no hemodynamic instability, chest wall rigidity, or ventilatory compromise was observed. The absence of complications in two patients does not establish safety, and these observations should not be interpreted as support for the dosing regimen in other patients.

These two cases describe a pharmacokinetically informed approach to emergence after prior severe EA. The uncontrolled clinical circumstances, modeled rather than measured drug Ces, and small number of observations preclude conclusions about efficacy, safety, or a specific Ce threshold. These findings are hypothesis‐generating: they do not establish that this strategy caused the calm emergences observed, but they support prospective study of whether separating the decline in propofol Ce from the time of awakening reliably reduces EA in patients with a prior episode. If confirmed in a controlled study, this pharmacokinetically guided approach could offer clinicians a practical strategy for managing patients at high risk of recurrent EA.

## Funding

This work received no specific funding and was performed as part of the authors’ routine clinical and academic duties at the University of Utah.

## Ethics Statement

The University of Utah Case Report Using Existing Data Author Worksheet indicated that IRB submission was not required.

## Conflicts of Interest

The authors declare no conflicts of interest.

## Supporting Information

Additional supporting information can be found online in the Supporting Information section.

## Supporting information


**Supporting Information** Care.

## Data Availability

The data supporting the findings of this case report, including the simulated pharmacokinetic values underlying Figures 1, 2, and 3, are available from the corresponding author upon reasonable request. No additional datasets were generated or analyzed.

## References

[1] Evered L. , Silbert B. , Knopman D. S. et al., Recommendations for the Nomenclature of Cognitive Change Associated With Anaesthesia and surgery-2018, British Journal of Anaesthesia. (2018) 121, no. 1, 1005–1012, 10.3233/jad-189004. PMC7069032 30336844

[2] Lepouse C. , Lautner C. A. , Liu L. , Gomis P. , and Leon A. , Emergence Delirium in Adults in the Post-Anaesthesia Care Unit, British Journal of Anaesthesia. (2006) 96, no. 6, 747–753, 10.1093/bja/ael094. 16670111

[3] Wei B. , Feng Y. , Chen W. , Ren D. , Xiao D. , and Chen B. , Risk Factors for Emergence Agitation in Adults After General Anesthesia: A Systematic Review and Meta-Analysis, Acta Anaesthesiologica Scandinavica. (2021) 65, no. 6, 719–729, 10.1111/aas.13774. 33370461

[4] Ham S. Y. , Kim J. E. , Park C. , Shin M. J. , and Shim Y. H. , Dexmedetomidine Does Not Reduce Emergence Agitation in Adults Following Orthognathic Surgery, Acta Anaesthesiologica Scandinavica. (2014) 58, no. 8, 955–960, 10.1111/aas.12379. 25132201

[5] Na H. S. , Kim S. Y. , Park J. I. , Lee S. , Hwang J. W. , and Shin H. J. , The Effect of Intranasal Dexmedetomidine Administration on Emergence Agitation or Delirium in Pediatric Patients After General Anesthesia: A Meta-Analysis of Randomized Controlled Trials, Paediatric Anaesthesia. (2023) 33, 636–646. 10.1111/pan.14689 37128675

[6] Johnson K. B. , Syroid N. D. , Gupta D. K. et al., An Evaluation of Remifentanil Propofol Response Surfaces for Loss of Responsiveness, Loss of Response to Surrogates of Painful Stimuli and Laryngoscopy in Patients Undergoing Elective Surgery, Anesthesia & Analgesia. (2008) 106, no. 2, 471–479, 10.1213/ane.0b013e3181606c62. PMC3050649 18227302

[7] Schnider T. W. , Minto C. F. , Gambus P. L. et al., The Influence of Method of Administration and Covariates on the Pharmacokinetics of Propofol in Adult Volunteers, Anesthesiology. (1998) 88, no. 5, 1170–1182, 10.1097/00000542-199805000-00006. 9605675

[8] Minto C. F. , Schnider T. W. , Egan T. D. et al., Influence of Age and Gender on the Pharmacokinetics and Pharmacodynamics of Remifentanil. I. Model Development, Anesthesiology. (1997) 86, no. 1, 10–23, 10.1097/00000542-199701000-00004. 9009935

[9] Lee S. J. and Sung T. Y. , Emergence Agitation: Current Knowledge and Unresolved Questions, Korean Journal of Anesthesiology. (2020) 73, no. 6, 471–485, 10.4097/kja.20097. PMC7714637 32209961

[10] Choi Y. H. , Kim K. M. , Lee S. K. et al., Effects of Remifentanil and Remifentanil-Alfentanil Administration on Emergence Agitation After Brief Ophthalmic Surgery in Children, BMC Anesthesiology. (2016) 16, no. 1, 10.1186/s12871-016-0213-2. PMC4969655 27484339

[11] Li J. , Zhu H. , Wang Y. , Chen J. , He K. , and Wang S. , Remifentanil is Superior to Propofol for Treating Emergence Agitation in Adults After General Anesthesia, Drug Design, Development and Therapy. (2024) 18, 341–350, 10.2147/dddt.s433155. PMC10859049 38344258

[12] Wang M. L. , Min J. , Sands L. P. , Leung J. M. , and Perioperative Medicine Research Group , Midazolam Premedication Immediately Before Surgery is Not Associated With Early Postoperative Delirium, Anesthesia & Analgesia. (2021) 133, no. 3, 765–771, 10.1213/ane.0000000000005482. PMC8373629 33721875

[13] De Cosmo G. , Sessa F. , Fiorini F. , and Congedo E. , Effect of Remifentanil and Fentanyl on Postoperative Cognitive Function and Cytokines Level in Elderly Patients Undergoing Major Abdominal Surgery, Journal of Clinical Anesthesia. (2016) 35, 40–46, 10.1016/j.jclinane.2016.07.016. 27871564

[14] Shin H. J. , Nam S. W. , Kim H. et al., Postoperative Delirium After Dexmedetomidine Versus Propofol Sedation in Healthy Older Adults Undergoing Orthopedic Lower Limb Surgery With Spinal Anesthesia: A Randomized Controlled Trial, Anesthesiology. (2023) 138, no. 2, 164–171, 10.1097/aln.0000000000004438. 36534899

