# Supplementary material for: Remifentanil to Maintain Unresponsiveness While Propofol Concentrations Decline Before Emergence in Two Patients With Prior Emergence Agitation
Source: Case Rep Anesthesiol. 2026 Nov 1;2026:9359809. doi: 10.1155/cria/9359809 (PMC13724829; doi:10.1155/cria/9359809)
Supplement: Supplementary file 1 [file CRIA-2026-9359809-s001.pdf]

CARE Checklist of information to include when writing a case report

CARE case report guidelines

(cc) BY-NC-ND

Topic	Item	Checklist item description	Reported on Line
Title	1	The diagnosis or intervention of primary focus followed by the words "case report"	1-2
Key Words	2	2 to 5 key words that identify diagnoses or interventions in this case report, including "case report"	42-43
Abstract (no references)	3a	Introduction: What is unique about this case and what does it add to the scientific literature?	29-31
	3b	Main symptoms and/or important clinical findings	32-36
	3c	The main diagnoses, therapeutic interventions, and outcomes	32-36
	3d	Conclusion—What is the main "take-away" lesson(s) from this case?	37-41
Introduction	4	One or two paragraphs summarizing why this case is unique (**may include references**)	45-64
Patient Information	5a	De-identified patient specific information.	68,107
	5b	Primary concerns and symptoms of the patient.	68-71,107-108
	5c	Medical, family, and psycho-social history including relevant genetic information	68,107
	5d	Relevant past interventions with outcomes	81-88,113-116
Clinical Findings	6	Describe significant physical examination (PE) and important clinical findings.	102-103,124-127
Timeline	7	Historical and current information from this episode of care organized as a timeline	67-130
Diagnostic Assessment	8a	Diagnostic testing (such as PE, laboratory testing, imaging, surveys).	78-80,98-101,124-127
	8b	Diagnostic challenges (such as access to testing, financial, or cultural)	N/A
	8c	Diagnosis (including other diagnoses considered)	81-84,114-116
	8d	Prognosis (such as staging in oncology) where applicable	N/A
Therapeutic Intervention	9a	Types of therapeutic intervention (such as pharmacologic, surgical, preventive, self-care)	91-105,119-123
	9b	Administration of therapeutic intervention (such as dosage, strength, duration)	97-102,119-123
	9c	Changes in therapeutic intervention (with rationale)	57-61,91-97,117-123
Follow-up and Outcomes	10a	Clinician and patient-assessed outcomes (if available)	104-105,128-130
	10b	Important follow-up diagnostic and other test results	78-80,98-101,124-127
	10c	Intervention adherence and tolerability (How was this assessed?)	102-103,126-127
	10d	Adverse and unanticipated events	81-88,113-116
Discussion	11a	A scientific discussion of the strengths AND limitations associated with this case report	180-192
	11b	Discussion of the relevant medical literature **with references**.	132-146
	11c	The scientific rationale for any conclusions (including assessment of possible causes)	169-174,184-192
	11d	The primary "take-away" lessons of this case report (without references) in a one paragraph conclusion	184-192
Patient Perspective	12	The patient should share their perspective in one to two paragraphs on the treatment(s) they received	Not included
Informed Consent	13	Did the patient give informed consent? Please provide if requested	Yes [X] No [ ]
